# Analysis of expression patterns of candidate genes for bud set and cold tolerance in scots pine (*Pinus sylvestris* l.)

**DOI:** 10.1186/1753-6561-5-S7-P3

**Published:** 2011-09-13

**Authors:** Komlan Avia, Kärkkäinen Katri, Outi Savolainen

**Affiliations:** 1 Department of Biology, University of Oulu, PO Box 3000, FI-90014 Oulu, Finland; 2 Finnish Forest Research Institute, FI-91500 Muhos, Finland

## 

Conifers, like other perennial plants adapt their growth rhythm to seasonal changes in the environment. Growth cessation and bud set are among the main adaptations used by the plants to prepare for winter. These adaptive traits are mainly controlled by photoperiod, temperature and light quality and show a clinal variation in different species including Scots pine. In conifers, efforts have been made to understand the genetic basis of these traits. In Norway spruce for instance, a *Flowering Locus T* homolog (*PaFT4*) has been shown to be implicated in the control of growth rhythm.

We previously evaluated genetic variation for timing of bud set and cold tolerance in a large association population of Scots pine (*Pinus sylvestris* L.) from Punkaharju, Southern Finland, along with five other populations used as control for clinal variation. To correlate this genetic variation with the expression pattern of some candidate genes already described in other species including conifers as involved in the control of bud set (e.g. FT4, PRR1, Gi, Myb) and cold tolerance (e.g. Aba responsive, DHN3, ERD, LEA), the Punkaharju population was used together with two others from Kolari (Northern Finland) and Poland. For photoperiod treatments, seedlings of eight families from each population were grown in a greenhouse under natural photoperiod conditions and in growth chambers under constant light for three months before transfer to three different photoperiod conditions (20 h/4 h, 17 h/7 h and 8 h/16 h light/dark). For cold treatments, the same families were grown in growth chambers under constant light for two and a half months and divided into two parts. The first part was directly submitted to the cold treatments (+4°C for one week followed by -5°C for 48 hours) while for the second part, seedlings were allowed to set buds under short day conditions (8 h light/16 h dark) before treatments. The results of this expression study will be described in relation to the phenotypic variance already observed.

